# Clinical and functional patient characteristics predict medical needs in older patients at risk of functional decline

**DOI:** 10.1186/s12877-020-1443-1

**Published:** 2020-02-21

**Authors:** Anne-Carina Scharf, Janine Gronewold, Christian Dahlmann, Jeanina Schlitzer, Andreas Kribben, Guido Gerken, Helmut Frohnhofen, Richard Dodel, Dirk M. Hermann

**Affiliations:** 1 Department of Neurology, University Hospital Essen, University of Duisburg-Essen, Hufelandstraße 55, 45147 Essen, Germany; 2 Nursing Headquarters, University Hospital Essen, University of Duisburg-Essen, Essen, Germany; 3 Department of Nephrology, Geriatric and Internal Medicine, Alfried Krupp Hospital Essen, Essen, Germany; 4 Department of Nephrology, University Hospital Essen, University Duisburg-Essen, Essen, Germany; 5 Department of Gastroenterology and Hepatology, Faculty of Medicine, University Hospital Essen, University of Duisburg-Essen, Essen, Germany; 6 Faculty of Health, Department of Medicine, University Witten-Herdecke, Witten, Germany; 7 Department of Geriatrics, University Hospital Essen, Essen, Germany

## Abstract

**Background:**

The rising number of older multimorbid in-patients has implications for medical care. There is a growing need for the identification of factors predicting the needs of older patients in hospital environments. Our aim was to evaluate the use of clinical and functional patient characteristics for the prediction of medical needs in older hospitalized patients.

**Methods:**

Two hundred forty-two in-patients (57.4% male) aged 78.4 ± 6.4 years, who were consecutively admitted to internal medicine departments of the University Hospital Essen between July 2015 and February 2017, were prospectively enrolled. Patients were assessed upon admission using the Identification of Seniors at Risk (ISAR) screening followed by comprehensive geriatric assessment (CGA). The CGA included standardized instruments for the assessment of activities of daily living (ADL), cognition, mobility, and signs of depression upon admission. In multivariable regressions we evaluated the association of clinical patient characteristics, the ISAR score and CGA results with length of hospital stay, number of nursing hours and receiving physiotherapy as indicators for medical needs. We identified clinical characteristics and risk factors associated with higher medical needs.

**Results:**

The 242 patients spent [median(Q1;Q3)]:9.0(4.0;16.0) days in the hospital, needed 2.0(1.5;2.7) hours of nursing each day, and 34.3% received physiotherapy.

In multivariable regression analyses including clinical patient characteristics, ISAR and CGA domains, the factors age (β = − 0.19, 95% confidence interval (CI) = − 0.66;-0.13), number of admission diagnoses (β = 0.28, 95% CI = 0.16;0.41), ADL impairment (B = 6.66, 95% CI = 3.312;10.01), and signs of depression (B = 6.69, 95% CI = 1.43;11.94) independently predicted length of hospital stay. ADL impairment (B = 1.14, 95%CI = 0.67;1.61), cognition impairment (B = 0.57, 95% CI = 0.07;1.07) and ISAR score (β =0.26, 95% CI = 0.01;0.28) independently predicted nursing hours. The number of admission diagnoses (risk ratio (RR) = 1.06, 95% CI = 1.04;1.08), ADL impairment (RR = 3.54, 95% CI = 2.29;5.47), cognition impairment (RR = 1.77, 95% CI = 1.20;2.62) and signs of depression (RR = 1.99, 95% CI = 1.39;2.85) predicted receiving physiotherapy.

**Conclusion:**

Among older in-patients at risk for functional decline, the number of comorbidities, reduced ADL, cognition impairment and signs of depression are important predictors of length of hospital stay, nursing hours, and receiving physiotherapy during hospital stay.

## Background

With the ongoing demographic changes, hospitals face a constantly rising number of older, often multimorbid patients. This has profound implications for patient care [1–4]. Older patients with multimorbidity are characterized by multidimensional impairments including physiological, emotional, social, and cognitive deficits, which are associated with higher risk of functional decline [3, 5–7]. Older patients with chronic and complex conditions are more vulnerable and likely to fall, and have a higher number of risk factors which could extend and complicate their hospital stay and exacerbate complications [1, 8]. Since the number of such patients at risk is expected to increase due to demographical changes, attempts have been made to identify these patients using screenings and assessments [9, 10]. By now the ISAR screening tool is one of the most commonly used tools to predict the risk of functional decline in older patients [6, 11, 12]. For high-risk patients with positive ISAR screening a comprehensive geriatric assessment (CGA) is recommended as a second diagnostic step. The ISAR screening in combination with the CGA has recently been validated for acute medical departments by Scharf et al. [13]. Since the CGA is a time- and resource consuming assessment it is not efficient to perform CGA in all patients [14, 15]. In patients at risk for functional decline, the CGA enables caregivers to collect further information about patients’ clinical and functional characteristics and offers a possibility to gain a better understanding of mechanisms underlying needs for intensified in-hospital medical care [16].

The need of intensified medical care in older patients as reflected by prolonged length of hospital stay, more nursing hours and patients receiving physiotherapy challenges personnel and financial resources [8, 17, 18]. Previous studies have shown that prolonged length of hospital stay is predicted by factors including female sex and polypharmacy in patients aged ≥65 years admitted to acute internal and geriatric wards [8] and by age ≥ 60 and higher number of comorbidities in colon cancer patients aged < 60 to > 80 years [19]. Thus far, it is unknown which patient characteristics predict prolonged length of hospital stay in older internal medicine in-patients who are at risk for functional decline defined by a positive ISAR.

An association between nursing hours and patients’ functional status seems obvious but systematic evidence-based analyses are still scarce [20]. A study conducted by Sousa et al. found that nursing hours in intensive care patients were predicted by higher age and severity of illness and it were higher in surgical patients than in patients in internal medicine wards [21]. In patients admitted to surgery wards, comorbidities, the ISAR score, mobility impairment, ADL impairment and cognition impairment were predictors of nursing hours [6]. Evidence for the prediction of nursing hours in internal medicine in-patients is still missing as is evidence for the prediction of physiotherapy, which reflects mobility impairment and also contributes to functional recovery, in internal medicine in-patients. Previous studies evaluated how physiotherapy influenced patients’ medical needs [22, 23]. The association of physiotherapy with preexisting impairment was much less studied [24, 25].

There have hitherto been very few studies exploring higher medical needs in older patients at risk for functional decline. To further improve the process by which greater medical care is granted to certain patients, we need to understand which patient characteristics are associated with patient’s needs for intensified medical care in hospital environments. In our last manuscript, we evaluated the diagnostic validity of the ISAR score and the CGA conducting cutoff- and sensitivity/specificity analyses [13]. We now focus on the clinical application of these tools and study how patient characteristics, the ISAR score and CGA results are associated with length of hospital stay, nursing hours, and receiving physiotherapy in older internal medicine patients.

## Methods

### Study cohort

**Fig. 1:**
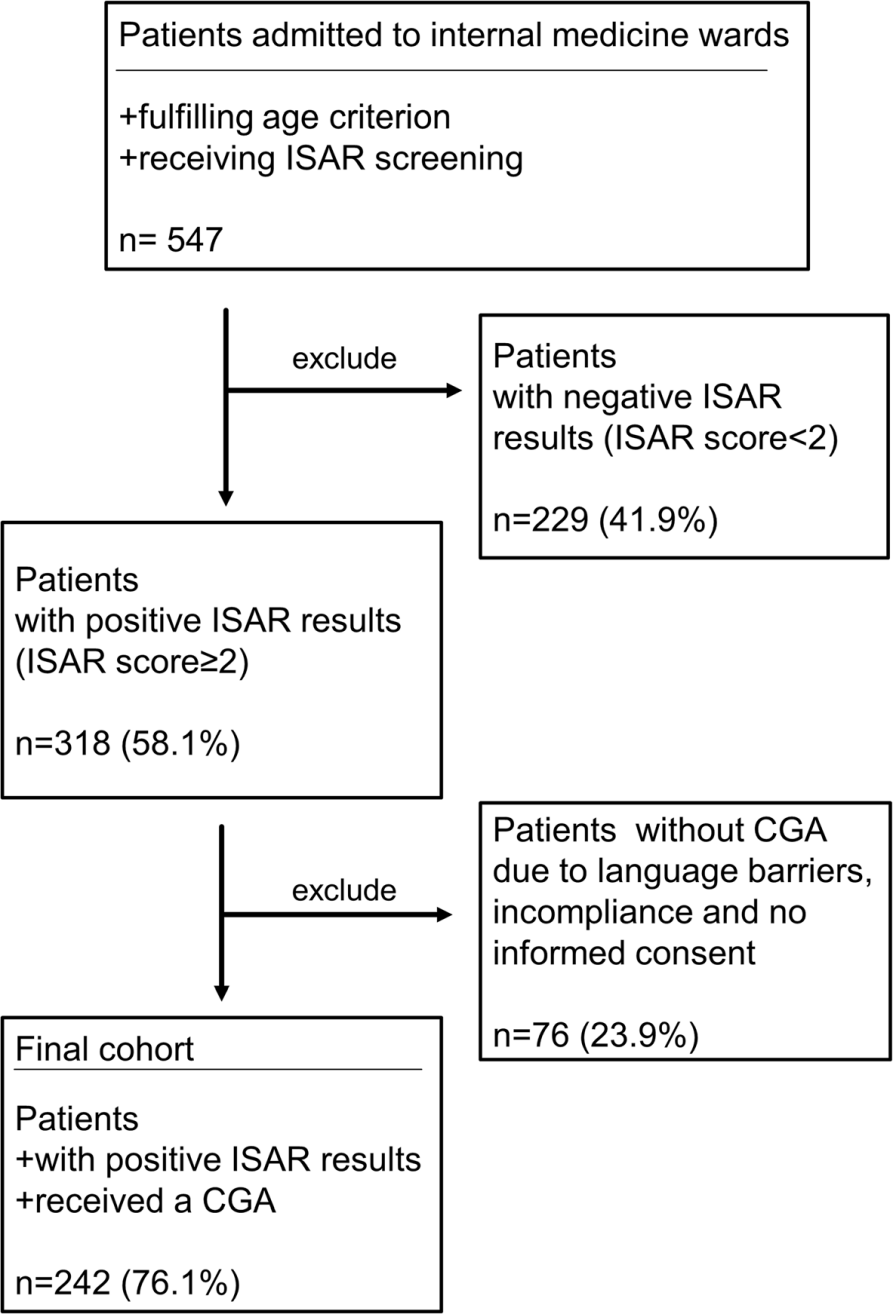
Flow chart of patients’ selection proces. (ISAR; Identification of Seniors at Risk, CGA; Comprehensive Geriatric Assessment)

The sample used for the present analyses included 242 hospitalized patients (57.2% male, mean ± standard deviation (SD) age 78.4 ± 6.4 years old) who were admitted electively or via emergency department to the internal medicine departments of the University Hospital Essen between July 2015 and February 2017 and who received ISAR screening by the nursing staff upon admission. Patients were included into the present study if they fulfilled an age criterion (see below) and received a positive ISAR screening (score ≥ 2) followed by CGA (Fig. 1) conducted by the nursing staff upon admission. The age criterion was a) ≥75 years for patients in the Department of Gastroenterology and Hepatology and in the Department of Cardiology and Angiology or b) ≥65 years for patients in the Department of Nephrology since nephrological patients exhibit premature aging [26, 27]. The CGA was performed within 3 days after admission by a geriatric liaison service (consisting of a geriatrician, a psychologist and an occupational therapist). The available data were obtained prospectively. More detailed information about study cohort characteristics and methodology have previously been reported (see [13]). The study was approved by the ethics committee of the University Duisburg-Essen and need for consent was waived.

### Clinical and functional characteristics of patients

#### Clinical characteristics

It has been proposed that admission diagnoses represent the best predictor for length of hospital stay [28]. Since we had a broad spectrum of different internal medicine diseases, we analyzed the number of admission diagnoses as an indicator of illness severity). Patients’ demographic data, diagnoses and medical histories were taken from the electronical Hospital Information System (HIS).

#### Functional characteristics: ISAR screening

We utilized a modified version of the original ISAR by McCusker [29]. This ISAR screening comprises six yes/no items about the following domains: Premorbid functional dependence, acute change in functional dependence within the last 24 h, hospitalization within the last 6 months, impaired vision, impaired memory and polypharmacy (≥6 medications). These items are summed up resulting in an ISAR score ranging from 0 to 6. An ISAR score ≥ 2 was interpreted as positive implying that patients with positive ISAR are at risk for functional decline.

#### Functional characteristics: CGA

The CGA included six commonly used geriatric tests. The Barthel Index was used for the assessment of ADL with a score ≤ 90 defined as impaired [30–32]. The Timed Up and Go [33] and the Tinetti Mobility Test [34] were used for the assessment of mobility. Mobility was rated as impaired if Timed Up & Go was ≥20 s and/or the patients had scores < 20 on the Tinetti Mobility Test [35, 36]. Cognition was assessed using the 30 item Mini-Mental State Examination (MMSE) [37] and the Clock-Drawing Test [38]. Cognition was interpreted as impaired if MMSE was ≤27 and/ or the clock-Drawing Test score was ≥3 [39, 40]. For the assessment of signs of depression, we applied the Geriatric Depression Scale (GDS). A GDS score ≥ 6 was interpreted as the presence of signs of depression [41].

### In-hospital medical needs

Measures of in-hospital medical needs comprised length of hospital stay in days, nursing hours per day and received physiotherapy (yes/no), which were retrieved from the electronical HIS. Length of hospital stay was defined as the number of days from admission to discharge from the ward. Prolonged length of hospital stay was defined as ≥7 days, which is the minimum duration of geriatric rehabilitation in Germany. Nursing hours were documented using the “Leistungserfassung in der Pflege” catalogue, a set of approximately 180 items covering all features of in-patient nursing care which is widely used in German-speaking countries [20]. Each item includes a time value, which is coded as the default value or adapted based on nursing effort (for further details see [6]). More nursing hours were defined as ≥2 h per day as this was the median in our cohort. Receiving physiotherapy was again operationalized using the HIS data. Since 159 (65.7%) patients did not receive physiotherapy, the variable was dichotomized into receiving physiotherapy and not receiving physiotherapy.

### Statistical analyses

Continuous variables were presented as mean ± SD for normally distributed data (age) or as median and interquartile range (Q1;Q3) when data was not normally distributed (all other variables). Categorical variables were shown as numbers and percentages (%). We determined the sample size using the sample size calculator G*Power [42] (see Additional file 1: supplemental material S1).

We dichotomized patients’ outcome variables into

- length of hospital stay <7 days vs ≥7 days,
- nursing hours above median (≥2 hours) vs below median (<2 hours), and
- receiving physiotherapy vs not receiving physiotherapy

To compare these characteristics between patients with low and high medical needs, we used t-tests for normally distributed continuous data, Mann-Whitney-U-tests for data that was not normally distributed and χ^2^ tests for categorical data.

To evaluate the predictors for length of hospital stay in days and nursing hours per day, unadjusted univariable and adjusted multivariable linear regressions (forced entry method) were calculated. Since only about one third of patients (34.3%) received physiotherapy during hospital stay, we used the dichotomized variable of receiving physiotherapy (yes vs no) in uni- and multivariable Poisson regressions with robust error variance. The factors age, sex, number of admission diagnoses, ISAR score, ADL, mobility impairment and cognition impairment as well as signs of depression were first inserted unadjusted into univariable linear and univariable Poisson regressions. In a next step we analyzed the effects of the following models on in-hospital medical needs (length of hospital stay, nursing hours, and receiving physiotherapy).

- Model 1 including the ISAR score adjusted for age and sex
- model 2 including CGA domains (mobility impairment, cognition impairment, signs of depression and ADL impairment) adjusted for age and sex,
- model 3 including CGA domains (as above) and the ISAR score adjusted for age and sex and
- model 4 including CGA domains (as above), ISAR score, and number of admission diagnoses adjusted for age and sex.

In regression analyses, missing data were excluded list-wise, while in the other calculations, cases were only excluded if outcome variables were missing. All analyses were performed using Statistical Package for Social Science 22 (SPSS 22) for Windows (SPSS, Chicago, IL, U.S.A.).

## Results

### Study cohort

The 242 patients of the total cohort (78.4 ± 6.4 years and 57.2% male) spent 9.0(4.0; 16.0) (median(Q1;Q3)) days in hospital, and received 2.0(1.5;2.7) hours of nursing each day. Approximately one third (34.3%) received physiotherapy. Of the total cohort, 48.8% had chronic kidney disease, 38.8% had cancer, and 39.7% had coronary heart disease. ADL impairment was present in 47.1%, mobility impairment in 35.1%, cognition impairment in 53.7%, and signs of depression in 11.6% of the total cohort. Further demographic and medical data including comorbidities for the total cohort and split by high and low medical needs are shown in Table 1 and Additional file 1: supplemental material S2.

**Table 1:** Characteristics of the total cohort also split by low vs high medical needs

	Total cohort (*n* = 242)	Length of hospital stay		Nursing hours per days		Received physiotherapy	
		< 7 days *n* = 100, 41.3%	≥7 days *n* = 142, 58.7%	< 2 h *n* = 104, 49.1%	≥2 h *n* = 108, 50.9%	No *n* = 159, 65.7%	Yes *n* = 83, 34.3%
Age (years), mean ± SD	78.41 ± 6.4	79.0 ± 5.9	78.04 ± 6.7	77.44 ± 6.0	78.87 ± 6.5	78. 13 ± 6.0	78.95 ± 7.0
Sex (male), n(%)	139 (57.2)	65 (65.0)*	74 (52.1)	67 (64.4)	56 (51.9)	100 (62.9)*	44 (53.0)
Number of admission diagnoses, (median[Q1;Q3])	1.0 [1.0;3.25]	1.0 [1.0;2.0 †	2.0 [2.0;6.0]	1.0 [1.0;6.0]	2.0 [1.0;3.0]	1.0 [1.0;2.0]†	3.0 [1.0;7.0]
ISAR (score), (median[Q1;Q3])	2.0 [2.0;4.0]	2.0 [2.0;3.0]	3.0 [2.0;4.0]	2.0 [2.0;3.0]*	3.0 [2.0;4.0]	2.0 [2.0;3.0]*	3.0 [2.0;4.0]
ADL impairment, n(%)	114 (47.1)	35 (35.0)*	79 (55.6)	35 (33.7)†	66 (61.1)	51 (32.1)†	63 (75.9)
Mobility impairment, n(%)	85 (35.1)	26 (26.0)*	59 (41.5)	31 (29.8)*	46 (42.6)	47 (29.6)*	38 (45.8)
Cognition impairment, n(%)	130 (53.7)	47 (47.0)	83 (58.5)	50 (48.1)	65 (60.2)	75 (47.2)*	55 (66.3)
Signs of depression, n(%)	28 (11.6)	6 (6.0)*	22 (15.5)	12 (11.5)	13 (12.0)	11 (6.9)*	17 (20.5)
Length of hospital stay (days), (median[Q1;Q3])	9.0 (4.0;16.0)	3.5 (3.0;5.0)†	14.0 (10.0;24.3)	8.0 (4.0;13.8)	11.0 (3.0;21.0)	6.0 (3.0;11.0)†	20.0 (11.0;30.0)
Nursing hours per day (hours/day), (median[Q1;Q3])	2.01 (1.5;2.7)	1.9 (1.4;2.6)†	2.1 (1.6;2.9)	1.5 (1.3;1.8)†	2.7 (2.3;3.3)	1.8 (1.4;2.5)*	2.6 (1.9;3.3)
Received physiotherapy, n(%)	83 (34.3)	7 (7.0)†	76 (53.5)	25 (24.0)*	48 (44.4)	0.0 (0.0)†	83 (100.0)
Arterial hypertension, n(%)	193 (79.8)	77 (77.0)	116 (81.7)	85 (81.7)	88 (81.5)	124 (78.0)	69 (83.1)
Hyperlipoproteinemia, n(%)	134 (55.4)	51 (51.0)	83 (58.5)	63 (60.6)	59 (54.6)	89 (56.0)	45 (54.2)
Diabetes	78 (32.2)	35 (35.0)	43 (30.3)	34 (32.7)	36 (33.3)	54 (34.0)	24 (28.9)
History of stroke	31 (12.8)	11 (11.0)	20 (14.1)	9 (8.7)	15 (13.9)	16 (10.1)	15 (18.1)
Dementia, n(%)	25 (10.3)	9 (9.0)	16 (11.3)	4 (3.8)*	14 (13.0)	14 (8.8)	11 (13.3)
History of myocardial infarction	22 (9.1)	9 (9.0)	13 (9.2)	10 (9.6)	7 (6.5)	13 (8.2)	9 (10.8)
Coronary heart disease	96 (39.7)	33 (33.0)	63 (44.4)	50 (48.1)	40 (37.0)	63 (39.6)	33 (39.8)
Valve insufficiency	92 (38.0)	33 (33.0)	59 (41.5)	40 (38.5)	39 (36.1)	57 (35.8)	35 (42.2)
Chronic obstructive pulmonary disease	32 (13.2)	12 (12.0)	20 (14.1)	18 (17.3)	10 (9.3)	17 (10.7)	15 (18.1)
Peripheral artery disease	43 (17.8)	14 (14.0)	29 (20.4)	23 (22.1)	16 (14.8)	29 (18.2)	14 (16.9)
Chronic kidney disease, n(%)	118 (48.8)	46 (46.0)	72 (50.7)	53 (51.0)	49 (45.4)	81 (50.9)	37 (44.6)
Cancer, n(%)	94 (38.8)	50 (50.0)*	44 (31.0)	46 (44.2)	34 (31.5)	73 (45.9)*	21 (25.3)
Depression, n(%)	12 (5.0)	5 (5.0)	7 (4.9)	1 (1.0)*	10 (9.3)	8 (5.0)	4 (4.8)

* *p* ≤ 0.05 or †*p* ≤ 0.001 compared to the corresponding low vs high medical needs; *ADL*, activities of daily living, *ISAR*, Identification of Seniors at Risk

### Factors associated with needs for intensified medical care

#### Length of hospital stay

##### Comparison of patients staying < 7 vs ≥7 days in hospital

142 (58.7%) of the patients stayed for ≥7 days in hospital. Compared with patients staying < 7 days (*n* = 100), patients who stayed for ≥7 days were significantly more often female (47.9% vs 35.0%, *p* = 0.046) had a higher number of diagnoses at admission (median(Q1;Q3) = 2.0(2.0;6.0) vs 1.0(1.0;6.0), *p* < 0.001), received more nursing hours (2.1(1.6;2.9) vs 1.9(1.4;2.6), *p* < 0.001), and more often received physiotherapy (53.5% vs 7.0%, *p* < 0.001). They were also more often impaired in the CGA domains ADL (55.6% vs 47.1%, *p* = 0.002), mobility (41.5% vs 26.0%, *p* = 0.012), signs of depression (15.5% vs 6.0%, *p* = 0.022) with a tendency towards significance in cognition (58.5% vs 47.0%, *p* = 0.071). Age and the ISAR score did not significantly differ between patients staying ≥7 days and patients staying < 7 days (Table 1).

##### Predictors of length of hospital stay in multivariable regression models

In unadjusted regression analyses, younger age (β = − 0.19, 95% CI = -0.66;-0.13), higher number of admission diagnoses (β = 0.28, 95% CI = 0.16;0.41), ADL impairment (B = 6.66, 95% CI = 3.31;10.01) and signs of depression (B = 6.69, 95% CI = 1.43;11.94) were significantly associated with longer hospital stay in the total cohort. In a multivariable regression including the ISAR score, age and sex, only younger age remained a significant predictor (model 1 in Table 2). Replacing the ISAR with CGA results, ADL impairment and cognition impairment as well as signs of depression were associated with longer hospital stay in addition to younger age (model 2 in Table 2). The addition of the ISAR score did not influence regression model characteristics to a relevant degree (model 3 in Table 2) whereas further adding the number of admission diagnoses (model 4 in Table 2) improved the regression model from R^2^ = 0.143 to 0.197. That is, because a higher number of admission diagnoses was a significant predictor of longer hospital stays, as were ADL impairment, cognition impairment, signs of depression and younger age.

**Table 2:** Predictors of length of hospital stay (in days)

	Unadjusted			Model 1 Corrected R^2^ = 0.038			Model 2 Corrected R^2^ = 0.147		
	β or B	95% CI	P	β or B	95% CI	P	β or B	95% CI	P
Age (years)	− 0.19	− 0.66;-0.13	0.004*	− 0.22	− 0.34;-0.09	0.001†	− 0.27	− 0.39;0.14	< 0.001†
Sex (male vs female)	1.203	−2.28;4.69	0.497	1.82	− 1.62;5.23	0.299	0.26	−3.05;3.52	0.876
Number of admission diagnoses	0.28	0.16;0.41	< 0.001†						
ISAR score	0.06	−0.26;0.72	0.363	0.104	− 0.02;0.23	0.108			
ADL impairment (yes vs no)	6.66	3.31;10.01	< 0.001†				8.03	4.43;11.88	< 0.001†
Mobility impairment (yes vs no)	1.47	−2.11;5.05	0.481				−2.08	−6.21;1.66	0.311
Cognition impairment (yes vs no)	3.35	−0.07;6.78	0.055				3.77	0.50;7.05	0.024*
Signs of depression (yes vs no)	6.69	1.43;11.94	0.013*				6.43	1.19;11.67	0.016*
				Model 3 Corrected R^2^ = 0.143			Model 4 Corrected R^2^ = 0.197		
				β or B	95% CI	P	β or B	95% CI	P
Age (years)				−0.27	−0.40;-0.14	< 0.001†	− 0.26	− 0.38;-0.14	< 0.001†
Sex (male vs female)				0.25	−3.05;3.56	0.880	0.04	−3.19;3.21	0.982
Number of admission diagnoses							0.24	0.12;0.36	< 0.001†
ISAR score				0.00	−0.13;0.13	0.966	0.00	−0.12;0.13	0.957
ADL impairment (yes vs no)				8.05	4.19;11.91	< 0.001†	7.38	3.72;11.26	< 0.001†
Mobility impairment (yes vs no)				−2.03	−5.97;1.91	0.312	−2.44	−6.46;1.19	0.210
Cognition impairment (yes vs no)				3.78	0.48;7.08	0.025*	3.72	0.50;6.89	0.023*
Signs of depression (yes vs no)				6.43	1.18;11.69	0.017*	5.85	0.78;10.95	0.025*

*ADL* activities of daily living, *ISAR* Identification of Seniors at Risk, *beta*, standardized regression coefficient, *B* unstandardized regression coefficient, *CI* confidence interval, *p ≤ 0.05 or † *p* ≤ 0.001

#### Nursing hours per day

##### Comparison of patients needing < 2 vs ≥2 h nursing per day

108 (50.9%) of the patients received ≥2 h of nursing per day. Compared with patients receiving < 2 h of nursing per day; patients with more nursing hours (≥2 h per day) more often had a diagnosis of dementia (13.0% vs 3.8%, *p* = 0.017), diagnosis of depression (9.3% vs 1.0%, *p* = 0.006), and diagnosis of pressure ulcer (11.1% vs 3.8%, *p* = 0.045. They also more often received physiotherapy (44.4% vs 24.0%, *p* = 0.002), had a higher ISAR score (3.0(2.0;4.0) vs 2.0(2.0;3.0), *p* = 0.002), and more often hadADL impairment (61.1% vs 33.7%, *p* < 0.001) and mobility impairment (42.6% vs 29.8%, *p* = 0.046). These two groups did not differ in age, number of admission diagnoses, and the CGA domains mobility, cognition, and signs of depression (Table 1).

##### Predictors of nursing hours in multivariable regression models

In unadjusted regression analyses, a higher ISAR score (β = 0.26, 95% CI = -0.01; 0.28), ADL impairment (B = 1.14, 95% CI = 0.67;1.61) and cognition impairment (B = 0.57, 95% CI = 0.07;1.07) were significant predictors of hours of nursing care received per day. In multivariable regression models, only ADL impairment remained a significant predictor (models 1–4 in Table 3).

**Table 3:** Predictors of nursing hours per day during hospital stay

	Unadjusted			Model 1 Corrected R^2^ = 0.022			Model 2 Corrected R^2^ = 0.051		
	β or B	95% CI	P	β or B	95% CI	P	β or B	95% CI	P
Age (years)	0.09	−0.05;0.23	0.196	0.05	−0.09;0.19	0.493	0.49	−1.95;0.22	0.578
Sex (male vs female)	0.42	−0.08;0.92	0.102	0.39	−0.11;0.89	0.121	0.19	−0.25;0.71	0.532
Number of admission diagnoses	0.03	−0.11;0.16	0.693						
ISAR score	0.26	0.01;0.28	0.032*	0.135	−0.01;0.27	0.056			
ADL impairment (yes vs no)	1.14	0.67;1.61	< 0.001†				1.01	0.95;2.09	0.008*
Mobility impairment (yes vs no)	0.31	−0.34;0.96	0.345				−0.35	−1.59;-0.38	0.393
Cognition impairment (yes vs no)	0.57	0.07;1.07	0.026*				0.38	−0.09;0.88	0.212
Signs of depression (yes vs no)	0.40	−0.37;1.17	0.311				−0.19	−0.50;1.04	0.727
				Model 3 Corrected R^2^ = 0.046			Model 4 Corrected R^2^ = 0.039		
				β or B	95% CI	P	β or B	95% CI	P
Age (years)				0.03	−0.12;0.21	0.519	0.05	−0.12;0.21	0.597
Sex (male vs female)				0.24	−0.41;0.82	0.513	0.21	−0.41;0.82	0.513
Number of admission diagnoses							−0.06	− 0.17;0.16	0.954
ISAR score				0.05	−0.13;0.21	0.669	0.04	−0.13;0.21	0.669
ADL impairment (yes vs no)				1.03	0.25;1.82	0.011*	1.03	0.24;1.83	0.011*
Mobility impairment (yes vs no)				−0.36	−1.59;-0.38	0.378	−0.36	−1.27;-0.45	0.379
Cognition impairment (yes vs no)				0.36	−0.26;0.96	0.252	0.36	−0.26;0.97	0.253
Signs of depression (yes vs no)				−0.19	−1.27;0.89	0.725	−0.19	−1.28;0.90	0.732

*ADL* activities of daily living, *ISAR* Identification of Seniors at Risk, *beta* standardized regression coefficient, *B* unstandardized regression coefficient, *CI* confidence interval, *p ≤ 0.05 or † *p* ≤ 0.001

#### Receiving physiotherapy

##### Comparison of patients receiving physiotherapy vs not receiving physiotherapy

83 patients (34.3%) received physiotherapy, whereas 159 patients did not. Compared with patients not receiving physiotherapy, patients receiving physiotherapy had pressure ulcers more often (12.0% vs 5.0%, *p* = 0.048). Patients who received physiotherapy stayed in hospital for longer (20.0 vs 6.0 days, *p* < 0.001), needed more hours of nursing (2.6(1.9;3.3) vs 1.8(1.4;2.5), *p* = 0.044), were female more often (47.0% vs 37.1%,*p* = 0.018) and had a higher ISAR score (3.0 (2.0;4.0), *p* = 0.040). Patients who received physiotherapy were also more often impaired in ADL (75.9% vs 32.1%, *p* < 0.001), mobility (45.8% vs 29.6%, *p* = 0.009), cognition (66.3% vs 47.2%, *p* = 0.029), more often showed signs of depression (20.5% vs 6.9%, *p* = 0.045) and had a higher number of admission diagnoses (3.0 (1.0;7.0) vs 1.0(1.0;2.0), *p* < 0.001). Age did not differ between these groups (Table 1).

##### Predictors of receiving physiotherapy in multivariable regression models

In unadjusted regressions, a higher number of admission diagnoses (RR = 1.06, 95% CI = 1.04;1.08), ADL impairment (RR = 3.54, 95% CI = 2.29;5.47), cognition impairment (RR = 1.77, 95% CI = 1.20;2.62), and signs of depression (RR = 1.99, 95% CI = 1.39;2.85) were significant predictors of receiving physiotherapy (Table 4). In a multivariable regression including age, sex and the ISAR score, only female sex remained a significant predictor (model 1 in Table 4). When replacing the ISAR with CGA results, ADL impairment and signs of depression were significantly associated with receiving physiotherapy (model 2 in Table 4). The addition of the ISAR score again did not influence regression model characteristics to a high degree (model 3 in Table 4). Further addition of the number of medical admission diagnoses (model 4 in Table 4) improved the regression model from R^2^ = 0.291 to 0.336. In addition to ADL impairment a higher number of admission diagnoses was a significant predictor of receiving physiotherapy with cognition impairment reaching significance now, as well. In contrast, signs of depression stayed slightly below the threshold of statistical significance (model 4 in Table 4) presumably because of a significant intercorrelation between number of admission diagnoses and signs of depression.

**Table 4:** Predictors of receiving physiotherapy during hospital stay

	Unadjusted			Model 1 Corrected R^2^ = 0.050			Model 2 Corrected R^2^ = 0.291		
	RR	95% CI	P	RR	95% CI	P	RR	95% CI	P
Age (years)	1.01	0.99;1.04	0.357	1.01	0.98;1.03	0.751	0.97	0.93;1.02	0.206
Sex (male vs female)	1.52	1.08;2.16	0.018*	1.52	1.07;2.14	0.018*	1.02	0.59;1.61	0.933
Number of admission diagnoses	1.06	1.04;1.08	< 0.001†						
ISAR score	1.17	1.00;1.36	0.050	1.16	0.99;1.35	0.053			
ADL impairment (yes vs no)	3.54	2.29;5.47	< 0.001†				3.16	1.76;5.67	< 0.001†
Mobility impairment (yes vs no)	1.44	0.84;2.46	0.186				0.69	0.39;1.22	0.206
Cognition impairment (yes vs no)	1.77	1.20;2.62	0.004*				1.69	0.96;2.97	0.070
Signs of depression (yes vs no)	1.99	1.39;2.85	< 0.001				1.86	1.03;3.37	0.039*
				Model 3 Corrected R^2^ = 0.291			Model 4 Corrected R^2^ = 0.336		
				RR	95% CI	P	RR	95% CI	P
Age (years)				0.97	0.93;1.02	0.203	0.98	0.93;1.02	0.288
Sex (male vs female)				1.04	0.62;1.74	0.896	1.05	0.62;1.77	0.854
Number of admission diagnoses							1.06	1.02;1.09	0.001
ISAR score				1.04	0.79;1.37	0.791	1.02	0.76;1.36	0.914
ADL impairment (yes vs no)				3.12	1.71;5.67	< 0.001†	2.86	1.57;5.20	0.001†
Mobility impairment (yes vs no)				0.69	0.39;1.22	0.198	0.75	0.42;1.32	0.315
Cognition impairment (yes vs no)				1.67	0.94;2.96	0.080	1.75	1.00;3.05	0.050
Signs of depression (yes vs no)				1.85	1.03;3.33	0.039*	1.75	0.96;3.20	0.070

*ADL* activities of daily living, *ISAR* Identification of Seniors at Risk, *CI* confidence interval, *RR* relative risk, * *p* ≤ 0.05 or † *p* ≤ 0.001

## Discussion

The present study identified predictors of medical needs represented by length of hospital stay, nursing hours and receiving physiotherapy in older hospitalized patients at risk for functional decline identified by a positive ISAR. In multivariable regressions, significant predictors of length of hospital stay were ADL impairment and cognition impairment as well as signs of depression, in addition to a higher number of admission diagnoses. Patients with more nursing hours (≥2 h) more often had a diagnosis of dementia and depression, as well as ADL impairment and mobility impairment than patients with < 2 h of nursing per day. Moreover, ADL impairment was a significant predictor of nursing hours per day in multivariable regressions. Predictors of receiving physiotherapy in multivariable regressions were a higher number of admission diagnoses and ADL impairment, whereas cognition impairment and signs of depression, were significant predictors in unadjusted univariate regression models.

### Comparison with literature

#### Length of hospital stay

In our study, the median length of hospital stay in the total cohort was 9.0 (4.0;16.0) days which is comparable to other older patient cohorts. The median stay in hospital was between 5 and 14 days in older patients admitted to geriatric and internal medicine wards [8, 17, 22, 43, 44]. In a previous study analyzing 419 patients aged ≥70 years from geriatric wards [28], higher age, number of admission diagnoses, incontinence, and ADL impairment predicted a longer hospital stay. In a cohort of older orthopedics and trauma surgery patients (82.5 ± 5.5 years) impairment of ADL, signs of depression, and a higher number of admission diagnoses predicted a longer hospital stay [6].

Available data concerning the predictive value of cognition impairment for length of hospital stay is ambiguous. Vetrano et al. showed that cognition impairment assessed by the MMSE did not predict the length of hospital stay in older patients (≥65 years) electively admitted to acute geriatric and internal medicine wards in Italy [8]. However, other studies showed that cognitive impairment or the diagnosis of dementia predicted a longer hospital stay [45, 46]. Binder and Robins showed that a lower MMSE score was a significant predictor of a longer hospital stay. A decline in the MMSE score over 1 year in community-dwelling older persons was associated with an higher risk of hospitalization and longer hospital stay (> 20 days) [47]. Cognitive impairment often remains undetected in hospitals. However, early identification of cognitive impairment while in the hospital is crucial since patients with cognitive impairment are often malnourished, have a greater risk of falls, higher mortality, longer hospital stay, and higher short-term readmission risk [48]. Besides cognition impairment, signs of depression were associated with a longer hospital stay in our study. In a meta-analysis, Jansen et al. described that patients with comorbid depression spent more days in hospital (mean 13.8 days) than patients without comorbid depression (mean 10.5 days) and that comorbid depression was also related to increased medical costs which was not further analyzed in their meta-analysis due to limited data [49].

#### Nursing

There is only limited research on how to predict nursing workload [50]. In a study with results comparable to the results of our study, conducted by Mueller et al. analyzing 50 geriatric patients in multivariable analyses, impairment of ADL measured by the Barthel Index was a highly significant predictor of nursing hours [20]. In a Canadian Multicenter study of Hall et al., hospitalized patients of internal medicine, as well as surgical and obstetric wards needed more nursing hours per day if they were older or if they suffered from more complex diseases, both of which were not associated with nursing hours in our cohort [51]. In our internal medicine patients, an impaired Barthel Index for the assessment of ADL was the only significant predictor of nursing workload and is therefore a useful tool for predicting older patients’ needs. One explanation for the influence of the Barthel Index in predicting nursing hours is that the Barthel Index is closely linked to the nursing anamnesis at the beginning of the nursing process, which includes planning how to address patients’ nursing needs and accordingly allocate nursing hours.

#### Physiotherapy

Receiving physiotherapy was also predicted by impairment in ADL and signs of depression in our internal medicine patients at risk for functional decline. Physicians prescribe physiotherapy for patients with ADL impairment because physiotherapy aims to restore the patients’ functional independence [52]. One explanation for the impact of depression could be that the prescription of physiotherapy was also based on the idea that the patient would benefit from physiotherapy because of its influence on mood [53]. Another explanation could be that depressed patients better vocalize their needs.

### Strengths and limitations

A major strength of this study was the prospective design and combination of clinical and functional patient characteristics, which allows the analysis of associations between clinical patient characteristics and the patients’ medical needs. We included a broad spectrum of internal medicine diseases and merged patients from cardiology, gastroenterology, and nephrology departments. By merging patients from different wards, we decreased the susceptibility of our data to local department specificities.

Since we only included internal medicine patients, our results should be transferred carefully to other medical specialties. In a German orthopedics and trauma surgery department, ADL impairment and signs of depression predicted length of hospital stay. Impairments in ADL and cognition and a higher ISAR score predicted nursing hours per day, and impairments in ADL and mobility predicted received physiotherapy [6]. This slightly different combination of predictors compared to our internal medicine patient cohort underline the importance of performing a full CGA that covers a variety of domains. Our study evaluated a group at particularly high risk for functional decline, which suffers from a broad range of medical problems. Further efforts will be needed to test the observations made in other medical environments [54, 55]. Our study evaluated medical needs in a university hospital environment. Further analyses are required to show if the identified risk factors can also predict medical needs in non-academic primary hospitals.

According to our sample size calculation (see Additional file 1: supplemental material S1), the cohort of 242 patients allowed us to perform regression analyses using eight predictors. Of the 318 patients evaluated for eligibility, a CGA could not be performed in 76 patients due a variety of reasons that comprised language barriers, lack of consent, or rapid hospital discharge. Therefore, our results may not be representative for patients with short hospital stays. The combination of clinical and functional patient characteristics was an additional strength of our study. We performed an extensive CGA in every patient which we combined with clinical routine data. Of course, CGA itself can influence medical care and change patients’ outcome e.g. by raising awareness of the patients’ medical needs and increasing the attention of the hospital staff. Using data of the HIS implies that data documentation is complete and adequate.

## Conclusion

Among older in-patients at risk for functional decline, the number of comorbidities, ADL impairment, cognition impairment, and signs of depression are important predictors of medical needs during hospital stay. Patients in needs of intensified medical care should be identified soon after admission. Their early identification enables appropriate care and treatment allocation.

## Supplementary information

**Additional file 1.** Supplement S1. Sample size calculation. Supplement S2. Characteristics of the total cohort also split by low vs high medical needs.

## Data Availability

All relevant data are included in the paper. If additional data is needed it can be made available via the ethics committee of the University Duisburg-Essen (ethikkommission@uk-essen.de) to researchers meeting the criteria for confidential data access.

## Abbreviations

ADL: Activities of daily living
CGA: Comprehensive geriatric assessment
CI: Confidence interval
GDS: Geriatric Depression Scale
HIS: Hospital information system
ISAR: Identification of Seniors at Risk screening
MMSE: Mini Mental State Examination
SD: Standard deviation
SPSS 22: Statistical Packing for Social Science 22
