# Supplementary material for: Clinical and functional patient characteristics predict medical needs in older patients at risk of functional decline
Source: BMC Geriatr. 2020 Feb 21;20:75. doi: 10.1186/s12877-020-1443-1 (PMC7035632; doi:10.1186/s12877-020-1443-1)
Supplement: Supplementary file 1 — Additional file 1. Supplement S1. Sample size calculation. Supplement S2. Characteristics of the total cohort also split by low vs high medical needs. [file 12877_2020_1443_MOESM1_ESM.docx]

## Supplement S1. Sample size calculation

We calculate the sample size using G*Power (http://www.psychologie.hhu.de/arbeitsgruppen/allgemeine-psychologie-und-arbeitspsychologie/gpower.html, [1], a software that helps to calculate the sample size needed when conducting a regression analysis. We used the effect size of a model predicting the total amount of physiotherapy, since studies showed that this outcome variable had the smallest effect size of CGA’s predictive value in multivariable regression analyses (R²=0.054 => f²=0.057) [2]. Using an effect size of f²=0.057, a significance level of α=0.05, a power of 1-β=0.95 and a number of 3 to 8 predictors, a sample size of n= 230 is determined for this study.

## Supplement S2. Characteristics of the total cohort also split by low vs high medical needs

|  | Total cohort (n=242) | Length of hospital stay | | Nursing hours per days | | Received physiotherapy | |
| --- | --- | --- | --- | --- | --- | --- | --- |
|  |  | <7 days  n=100, 41,3% | ≥7 days  n=142, 58,7% | <2 hours  n=104 | ≥2 hours  n= 108 | No  n=159 | Yes  n=83 |
| Age (years), mean±SD | 78.41±6.4 | 79.0± 5.9 | 78.04±6.7 | 77.44±6.0 | 78.87±6.5 | 78. 13±6.0 | 78.95±7.0 |
| Sex (male), n (%) | 139 (57.2) | **65 (65.0) *** | 74 (52.1) | 67 (64.4) | 56 (51.9) | **100 (62.9) *** | **44 (53.0)** |
| Number of admission diagnoses, (median [Q1; Q3]) | 1.0 [1.0;3.25] | **1.0 [1.0;2.0] †** | **2.0 [2.0;6.0]** | 1.0 [1.0;6.0] | 2.0 [1.0;3.0] | **1.0 [1.0;2.0] †** | **3.0 [1.0;7.0]** |
| ISAR (score), (median [Q1; Q3]) | 2.0 [2.0;4.0] | 2.0 [2.0;3.0] | 3.0 [2.0;4.0] | **2.0 [2.0;3.0] *** | **3.0 [2.0;4.0]** | **2.0 [2.0;3.0] *** | **3.0 [2.0;4.0]** |
| ADL impairment, n (%) | 114 (47.1) | **35 (35.0) *** | **79 (55.6)** | **35 (33.7) †** | **66 (61.1)** | **51 (32.1) †** | **63 (75.9)** |
| Mobility impairment, n (%) | 85 (35.1) | **26 (26.0) *** | **59 (41.5)** | **31 (29.8) *** | **46 (42.6)** | **47 (29.6) *** | **38 (45.8)** |
| Cognition impairment, n (%) | 130 (53.7) | 47 (47.0) | 83 (58.5) | 50 (48.1) | 65 (60.2) | **75 (47.2) *** | **55 (66.3)** |
| Signs of depression, n (%) | 28 (11.6) | **6 (6.0) *** | **22 (15.5)** | 12 (11.5) | 13 (12.0) | **11 (6.9) *** | **17 (20.5)** |
| Length of hospital stay  (days), (median [Q1; Q3]) | 9.0 (4.0;16.0) | **3.5 (3.0;5.0) †** | **14.0 (10.0;24.3)** | 8.0 (4.0;13.8) | 11.0 (3.0;21.0) | **6.0 (3.0;11.0) †** | **20.0 (11.0;30.0)** |
| Nursing hours per day  (hours/day), (median [Q1; Q3]) | 2.01 (1.5;2.7) | **1.9 (1.4;2.6) †** | **2.1 (1.6;2.9)** | **1.5 (1.3;1.8) †** | **2.7 (2.3;3.3)** | **1.8 (1.4;2.5) *** | **2.6 (1.9;3.3)** |
| Received physiotherapy, n (%) | 83 (34.3) | **7 (7.0) †** | **76 (53.5)** | **25 (24.0) *** | **48 (44.4)** | **0.0 (0.0) †** | **83 (100.0)** |
| Arterial hypertension, n (%) | 193 (79.8) | 77 (77.0) | 116 (81.7) | 85 (81.7) | 88 (81.5) | 124 (78.0) | 69 (83.1) |
| Hyperlipoproteinemia, n (%) | 134 (55.4) | 51 (51.0) | 83 (58.5) | 63 (60.6) | 59 (54.6) | 89 (56.0) | 45 (54.2) |
| Diabetes | 78 (32.2) | 35 (35.0) | 43 (30.3) | 34 (32.7) | 36 (33.3) | 54 (34.0) | 24 (28.9) |
| Nicotine abuse | 36 (14.9) | 12 (12.0) | 24 (16.9) | 19 (18.3) | 16 (14.8) | 25 (15.7) | 11 (13.3) |
| Obesity | 52 (21.5) | 20 (20.0) | 32 (22.5) | 18 (17.3) | 27 (25.0) | 33 (20.8) | 19 (22.9) |
| Anemia, n (%) | 80 (33.1) | 33 (33.1) | 47 (32.9) | 33 (31.7) | 35 (32.4) | 51 (32.1) | 29 (34.9) |
| Cataract, n (%) | 19 (7.9) | 8 (8.0) | 11 (7.7) | 8 (7.7) | 9 (8.3) | 12 (7.5) | 7 (8.4) |
| Presbyacusis | 11 (4.5) | 2 (2.0) | 9 (6.3) | 6 (5.8) | 3 (2.8) | 5 (3.1) | 6 (7.2) |
| History of stroke | 31 (12.8) | 11 (11.0) | 20 (14.1) | 9 (8.7) | 15 (13.9) | 16 (10.1) | 15 (18.1) |
| Dementia, n (%) | 25 (10.3) | 9 (9.0) | 16 (11.3) | **4 (3.8) *** | **14 (13.0)** | 14 (8.8) | 11 (13.3) |
| History of myocardial infarction | 22 (9.1) | 9 (9.0) | 13 (9.2) | 10 (9.6) | 7 (6.5) | 13 (8.2) | 9 (10.8) |
| Heart failure | 51 (21.1) | 20 (20.0) | 31 (21.8) | 26 (25.0) | 21 (19.4) | 36 (22.6) | 15 (18.1) |
| Coronary heart disease | 96 (39.7) | 33 (33.0) | 63 (44.4) | 50 (48.1) | 40 (37.0) | 63 (39.6) | 33 (39.8) |
| Atrial fibrillation | 84 (34.7) | 30 (30.0) | 54 (38.0) | 32 (30.8) | 43 (39.8) | 49 (30.8) | 35 (42.2) |
| Other cardiac arrhythmias | 35 (14.5) | 16 (16.0) | 19 (13.4) | 11 (10.6) | 17 (15.7) | 20 (12.6) | 15 (18.1) |
| Valve insufficiency | 92 (38.0) | 33 (33.0) | 59 (41.5) | 40 (38.5) | 39 (36.1) | 57 (35.8) | 35 (42.2) |
| Chronic obstructive pulmonary disease | 32 (13.2) | 12 (12.0) | 20 (14.1) | 18 (17.3) | 10 (9.3) | 17 (10.7) | 15 (18.1) |
| History of pulmonary embolism | 7 (2.9) | 1 (1.0) | 6 (4.2) | 5 (4.8) | 2 (1.9) | 4 (2.5) | 3 (3.6) |
| History of thrombosis | 22 (9.1) | 8 (8.0) | 14 (9.9) | 9 (8.7) | 12 (11.1) | 14 (8.8) | 8 (9.6) |
| Peripheral artery disease | 43 (17.8) | 14 (14.0) | 29 (20.4) | 23 (22.1) | 16 (14.8) | 29 (18.2) | 14 (16.9) |
| Chronic kidney disease, n (%) | 118 (48.8) | 46 (46.0) | 72 (50.7) | 53 (51.0) | 49 (45.4) | 81 (50.9) | 37 (44.6) |
| Cancer, n (%) | 94 (38.8) | **50 (50.0) *** | **44 (31.0)** | 46 (44.2) | 34 (31.5) | **73 (45.9) *** | **21 (25.3)** |
| Parkinson’s disease | 2 (0.8) | 0 (0.0) | 2 (1.4) | 1 (1.0) | 1 (0.9) | 2 (1.3) | 0 (0.0) |
| Polyneuropathy | 24 (9.9) | 13 (13.0) | 11 (7.7) | 9 (8.7) | 14 (13.0) | 18 (11.3) | 6 (7.2) |
| Rheumatism | 9 (3.7) | 4 (4.0) | 5 (3.5) | 5 (4.8) | 4 (3.7) | 5 (3.1) | 4 (4.8) |
| Hyperthyroidism | 6 (2.5) | 3 (3.0) | 3 (2.1) | 4 (3.8) | 2 (1.9) | 3 (1.9) | 3 (3.6) |
| Hypothyroidism | 41 (16.9) | 16 (16.0) | 25 (17.6) | 19 (18.3) | 15 (13.9) | 26 (16.4) | 15 (18.1) |
| Alcohol abuse, n (%) | 9 (3.7) | **7 (7.0) *** | **2 (1.4)** | 6 (5.8) | 3 (2.8) | 8 (5.0) | 1 (1.2) |
| Depression, n (%) | 12 (5.0) | 5 (5.0) | 7 (4.9) | **1 (1.0) *** | **10 (9.3)** | 8 (5.0) | 4 (4.8) |
| Anxiety disorder | 0 (0.0) | 0 (0.0) | 0 (0.0) | 0 (0.0) | 0 (0.0) | 0 (0.0) | 0 (0.0) |
| Pressure ulcers, n (%) | 18 (7.4) | 4 (4.0) | 14 (9.9) | **4 (3.8) *** | **12 (11.1)** | **8 (5.0) *** | **10 (12.0)** |
| Anal incontinence | 2 (0.8) | 2 (2.0) | 0 (0.0) | 1 (1.0) | 1 (0.9) | 2 (1.3) | 0 (0.0) |
| Urinary incontinence | 2 (0.8) | 0 (0.0) | 2 (1.4) | 0 (0.0) | 2 (1.9) | 1 (0.6) | 1 (1.2) |

* p≤0.05 or †p≤0.001 compared to the corresponding low vs high medical needs; ADL, activities of daily living; ISAR, Identification of Seniors at Risk

## References

1. Faul F, Erdfelder E, Buchner A, Lang A-G: **Statistical power analyses using G*Power 3.1: Tests for correlation and regression analyses**. *Behavior Research Methods* 2009, **41**(4):1149-1160.

2. Gronewold J, Dahlmann C, Jäger M, Hermann DM: **Identification of hospitalized elderly patients at risk for adverse in-hospital outcomes in a university orthopedics and trauma surgery environment**. *PloS one* 2017, **12**(11):e0187801.
